# A positive feedback loop regulation between NOTCH1 and USP11 in T-cell leukemia

**DOI:** 10.1038/s41375-023-02096-4

**Published:** 2023-11-25

**Authors:** Igor Fijalkowski, Jin Wang, Qi Jin, Jolien Van Laere, Valentina Serafin, John D. Crispino, Panagiotis Ntziachristos

**Affiliations:** 1 Department of Biomolecular Medicine, Ghent University, Ghent, Belgium; 2 Center for Medical Genetics, Ghent University and University Hospital, Ghent, Belgium; 3 Cancer Research Institute Ghent (CRIG), Ghent, Belgium; 4 Health Management Center, Renmin Hospital of Wuhan University, Wuhan, China; 5 Department of Biochemistry and Molecular Genetics, Northwestern University, Chicago, IL USA; 6 Division of Experimental Hematology, St. Jude Children’s Research Hospital, Memphis, TN USA; 7 Pediatric Research Institute Foundation “Città Della Speranza”, Padova, Italy; 8 Cellular Signaling Unit, Department of Biomedical, Metabolic and Neural Sciences, University of Modena and Reggio Emilia, Modena, Italy

**Keywords:** Leukaemia, Translational research

## To the Editor:

NOTCH1 is a transmembrane receptor in a highly conserved signaling pathway that controls cell fate decisions in various organisms and tissues. In contrast, its dysregulation is associated with developmental disorders transformation [1]. NOTCH1 is composed of extracellular and intracellular domains. Ligand binding of NOTCH1 extracellular domain leads to two consecutive cleavage steps mediated by ADAM-family metalloproteases and γ-secretase respectively, to generate active NOTCH1 intracellular domain, which orchestrated with other transcriptional coactivators, activates downstream gene expression. Notably, gain of function NOTCH1 mutations have been reported in more than 60% of T cell lymphoblastic leukemia (T-ALL) cases. Therefore, the dissection of the molecular mechanisms controlling NOTCH1 signaling might provide novel therapeutic windows.

>600 ubiquitin E3 ligases regulate ubiquitination and can be reversed by about 100 deubiquitinating (DUB) enzymes [2]. Previous studies highlighted the critical role of deubiquitination mediated by ubiquitin-specific proteases (USP) in stabilizing NOTCH1 both in the context of T-ALL or angiogenic sprouting [3, 4]. We recently demonstrated the role of USP7 in deubiquitinating and stabilizing NOTCH1 protein levels in this disease [4] and the role of deubiquitination in therapy resistance [5]. Survival data from the pediatric cancer genome project (PeCan) coupled with the expression levels of 52 USP family members showed that USP11 levels are associated with poor prognosis [5]. Protein levels of USP11 are significantly elevated in diagnostic samples of high-risk disease cases, suggesting it might be associated with resistance to therapy [5]. To better demonstrate the function of USP11 in regulating NOTCH1 signaling, we utilized biochemistry and molecular assays coupled with bioinformatic analysis of cancer datasets to reveal a novel positive feedback loop between NOTCH1 and USP11 in T-ALL.

**Fig. 1:**
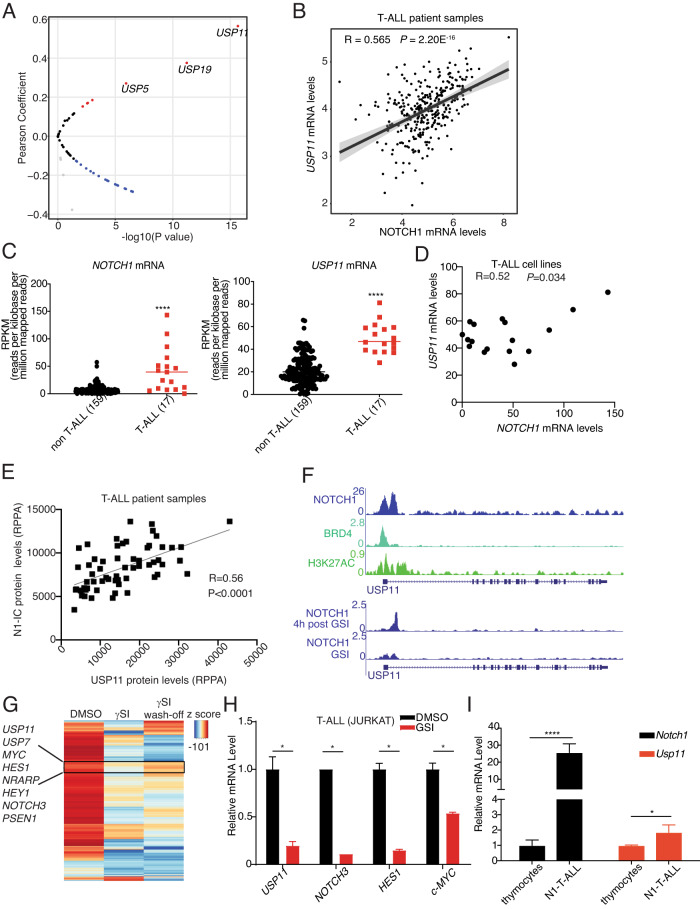
NOTCH1 and USP11 expression levels are positively correlated in T-ALL. **A** Volcano plot showing Pearson correlation between 52 *USPs* and *NOTCH1* mRNA levels in T-ALL patient samples analyzed from the Pediatric Cancer Genome Project data portal (PeCan, St. Jude, Memphis). **B** Pearson correlation between *NOTCH1* and *USP11* mRNA levels in T-ALL patient samples (source: PeCan). **C** RPKM values for *NOTCH1* and *USP11* in 176 blood cancer cell lines were obtained from https://software.broadinstitute.org/morpheus/, using the CCLE RNA sequencing data. These include 17 T-ALL cell lines, and all other cell lines were analyzed against these T-ALL cell lines. A two-tailed unpaired t-test was conducted using the RPKM values (****, *P* < 0.0001). **D** Pearson correlation between *NOTCH1* and *USP11* mRNA levels in 17 T-ALL cell lines analyzed from CCLE RNA sequencing data. **E** Reverse-phase protein array (RPPA) for USP11 and intracellular NOTCH1 (N1IC) protein levels (*n* = 61). **F** Tracks showing NOTCH1, BRD4, and the activating histone marks H3K27Ac ChIP-Seq signal enrichment in T-ALL cells (CUTLL1) at the *USP11* locus. **G** Heatmap representation of significant gene expression changes upon treatment of CUTLL1 cells with gamma-secretase inhibitor (γSI) followed by drug wash-off for 320’ (*wash-off*) [7]. Classical NOTCH1 targets (e.g., *HES1*, *MYC*, *NRAPR*) and the deubiquitinases *USP7* and *USP11* follow the intracellular NOTCH1 levels. **H** JURKAT T-ALL cells were treated with γSI (1 µM) for 24 h. RT-qPCR analysis of *USP11* and other NOTCH1 targets was shown (*P < 0.05). **I** RT-qPCR analysis of *Usp11* and *Notch1* in normal mouse thymocytes and spleen cells isolated from N1ΔE induced mouse T-ALL model (*P < 0.05, ****P < 0.00001).

NOTCH1 expression is frequently activated via mutation, transcriptional, and posttranslational regulation in T-ALL [1]. USP7 and NOTCH1 present with a feed-forward loop, with NOTCH1 transcriptionally starting the *USP7* locus [4]. We first analyzed the pediatric cancer genome project (PeCan) database to determine the correlation between the 52 USPs and NOTCH1 at the mRNA level (Fig. 1A). Among those 52 USPs, *USP11* was identified as transcript showing positive correlation with *NOTCH1* transcript (Fig. 1A, B). Further analysis of the Cancer Cell Line Encyclopedia (CCLE) database showed that both *NOTCH1* and *USP11* were found to be collectively highly expressed in T-ALL cell lines compared with cell lines from other cancer types (Fig. 1C), and their levels present with a positive correlation in the T-ALL group (Fig. 1D). To further confirm the positive correlation between NOTCH1 and USP11 expression, we performed reverse-phase protein array (RPPA) analysis in T-ALL patient samples. Our analysis shows that NOTCH1 protein levels positively correlate with USP11 protein levels (Fig. 1E). Together, these results demonstrate a positive correlation between USP11 and NOTCH1 expression in T-ALL.

Next, we questioned whether *NOTCH1* transcriptionally regulates USP11. First, we analyzed chromatin immunoprecipitation coupled to next-generation sequencing (ChIP-seq) data of NOTCH1, BRD4, and H3K27Ac on the *USP11* locus in T-ALL cells. Of note, NOTCH1 peaks were identified on the *USP11* promoter associated with the H3K27Ac signal suggesting the potential transcriptional regulation of USP11 by NOTCH1 (Fig. 1F). Furthermore, γ-secretase inhibitor (γSI)-mediated inhibition of NOTCH1 [6] impaired the binding of NOTCH1 on *USP11* promoter whereas washing off the γSI after 4 h recovered NOTCH1 binding on *USP11* promoter suggesting the dynamic nature of NOTCH1 binding on *USP11* promoter [6] (Fig. 1F). In line with the ChIP-seq data, NOTCH1 inhibition led to a decrease of USP11 expression, followed by a rebound of USP11 expression upon drug wash off [7] (Fig. 1G). Similarly to CUTLL1 cells, treatment of JURKAT T-ALL cells with γSI also decreased *USP11* expression (Fig. 1H). To further confirm the transcriptional regulation of USP11 by NOTCH1, we employed a T-ALL mouse model induced by ectopic expression of the intracellular part of NOTCH1 in hematopoietic progenitors coupled to transplantation into lethally irradiated recipients. *USP11* mRNA levels were significantly higher in mouse T-ALL than in thymocytes (Fig. 1I). Taken together, we demonstrate transcriptional activation of *USP11* by NOTCH1 in T-ALL.

**Fig. 2:**
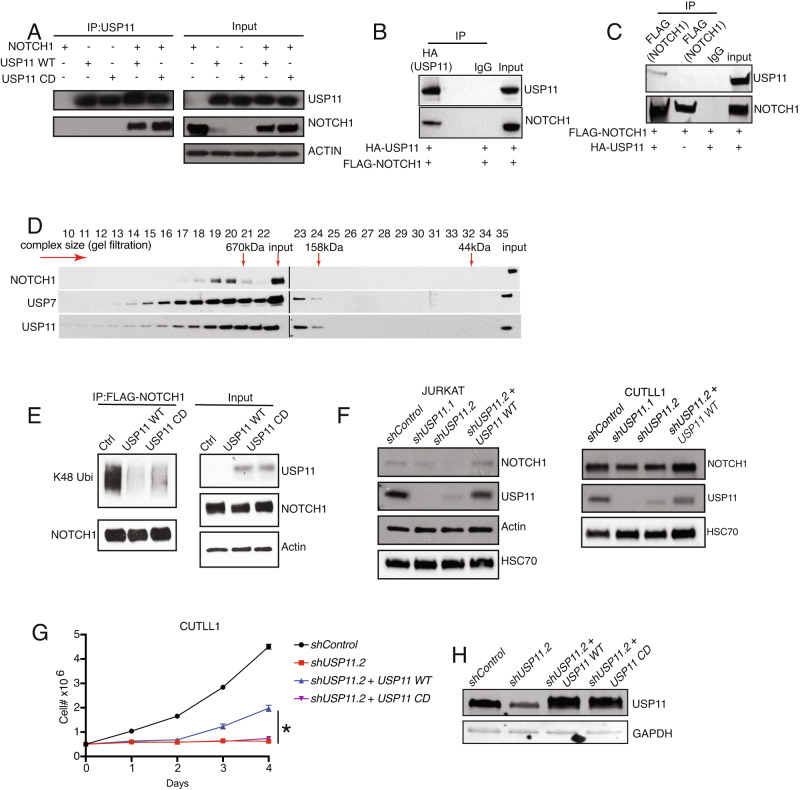
USP11 is a NOTCH1 deubiquitinase. **A** 293 T cells were transfected with the indicated plasmids for 48 h. Immunoblot studies after USP11 immunoprecipitation showing NOTCH1, USP11, and Actin. USP11 WT: catalytically active USP11; USP11 CD: catalytically inactive USP11. **B** 293 T cells were transfected with FLAG-tagged NOTCH1 and HA-tagged USP11 expressing constructs for 48 h coupled to immunoprecipitation using HA and IgG (control) antibodies. Immunoblot results for the detection of NOTCH1 and USP11 are shown. **C** Immunoblots for 293 T cells transfected with FLAG-tagged NOTCH1 only or FLAG-tagged NOTCH1 and HA-tagged USP11 for 48 h coupled to immunoprecipitation using FLAG and IgG (control) antibodies. Immunoblot results for the detection of NOTCH1 and USP11 are shown. In A-C, 10% of the lysate volume used in the IP reaction was loaded as input to indicate the protein size and amount in whole cell lysates. **D** Immunoblots studies for NOTCH1, USP7, and USP11 following isolation of whole-cell extracts and gel filtration chromatography in JURKAT cells. Fractions containing high to low molecular weight complexes (left to right) were run on a gradient 4–15% polyacrylamide gel. **E** 293T cells were transfected with FLAG-NOTCH1, ubiquitin, and FBXW7 associated with USP11 WT or USP11 CD as indicated. Immunoblots studies following immunoprecipitation of Flag-NOTCH1 in 293 T cells at denaturing condition. USP11 WT: catalytically active USP11; USP11 CD: catalytically inactive USP11. **F** JURKAT (left), or CUTLL1 (right) cells were transduced with indicated lentiviral vectors. Immunoblot studies show protein levels of NOTCH1, USP11, Actin, and HSC70. **G** Growth study over 4 days of wild-type (WT) and catalytically inactive (CD) USP11-expressing CUTLL1 cells transduced with *control* or *shUSP11.2* lentivirus (*n* = 3 biological replicates, *P ≤ 0.05). **H** Immunoblot detection of USP11 and GAPDH protein levels (day 0) in cells from (**G**).

We then hypothesized that USP11 might control NOTCH1 protein levels through deubiquitination based on the positive correlation between USP11 and NOTCH1 protein expression and the deubiquitinating activity of USP11. To address this, we first performed a co-immunoprecipitation assay by ectopic expression of USP11 and the intracellular part of NOTCH1 protein in 293 T cells. Immunoprecipitation studies showed that both the catalytically active and inactive forms of USP11 interact with NOTCH1, suggesting the binding of USP11 and NOTCH1 is independent of USP11 deubiquitinase activity (Fig. 2A, B). Immunoprecipitation of Flag-tagged NOTCH1 confirmed its interaction with USP11 protein (Fig. 2C). Our previous study demonstrated that USP7 interacts with NOTCH1 and controls leukemia growth by stabilizing the levels of NOTCH1 [4]. NOTCH1 interactome analysis in T-ALL cells has identified both USP7 and USP11 as interactors, and we have recently demonstrated that USP7 and USP11 form a complex in T-ALL [5, 8]. In agreement with this published evidence, gel filtration assay supported previous evidence that these proteins might exist in the same complex (Fig. 2D). USP7 deubiquitinates the Ankyrin domain of NOTCH1 [4]. As USP7 and USP11 form a complex in T-ALL, future studies are warranted to dissect further the detailed contribution of USP7 and USP11 to NOTCH1 posttranslational regulation and stabilization.

We then sought to determine whether USP11 could deubiquitinate NOTCH1 via ubiquitination assays. Ectopic expression of wild-type (WT) USP11 decreased the ubiquitination of NOTCH1, whereas USP11 catalytic inactive form partially rescued the ubiquitination of NOTCH1 in 293 T cells (Fig. 2E). We demonstrated that USP11 deubiquitinates NOTCH1. A previous study showed the potential interaction of USP11 and NOTCH1 in T-ALL cells, suggesting this regulation of NOTCH1 deubiquitination by USP11 might exist in T-ALL as well [8]. Deubiquitination is a posttranslational modification that controls functions such as protein degradation and stability, cellular localization, and protein-protein interaction. To further understand the potential regulation of NOTCH1 stability by USP11 in T-ALL, we induced *USP11* silencing by using two short hairpin RNAs (sh*USP11*) in T-ALL cells. We observed a decrease in NOTCH1 protein expression (Fig. 2F). Furthermore, decreased NOTCH1 expression via application of sh*USP11.2*, which targets the UTR region of *USP11*, in T-ALL cells could be rescued by ectopic expression of USP11, suggesting that regulation of NOTCH1 expression is USP11 dependent (Fig. 2G, and Supplementary Fig. 1).

Consistent with the rescue of NOTCH1 expression, ectopic expression of the USP11 WT, but not of the catalytically inactive form of USP11, partially rescued the proliferation inhibition induced by silencing of *USP11* in T-ALL cells (Fig. 2H, and Supplementary Fig. 1). Taken together, we identified a feedback loop regulation between USP11 and NOTCH1, in which NOTCH1 transcriptionally activates *USP11* expression and, in turn, USP11 controls NOTCH1 protein levels via deubiquitination.

Here, we identified a regulatory feedback loop between NOTCH1 and the USP11 deubiquitinase in T-cell acute lymphoblastic leukemia (T-ALL). NOTCH1 transcriptionally controls the *USP11* locus, whereas USP11 posttranslationally stabilizes NOTCH1 protein, leading to a positive correlation between NOTCH1 and USP11 mRNA and protein levels in T-ALL patients. Chromatin analyses demonstrated NOTCH1 binding on the *USP11* promoter. Modulation of NOTCH1 intracellular levels impacts its chromatin binding and reflects on *USP11* levels in a dynamic fashion. In turn, USP11 controls NOTCH1 expression through deubiquitination, potentially in a complex with another deubiquitinase, USP7. Further research is warranted to further explore the exact contribution of the catalytic versus non-catalytic (structural) roles of USP11 in the NOTCH1 complexes, as well as USP11 activities in conjunction with other deubiquitinating enzymes, such as USP7, and E3 ligases, e.g., FBXW7, that might interact with USP11 and NOTCH1 and are critical for NOTCH1 regulation [1, 4]. Potential roles for oncogenic transcription factors additional to NOTCH1 in USP11 regulation should be evaluated in the future. High expression of USP11 is associated with poor prognosis of colorectal and breast cancer. Increased USP11 levels can promote the in vitro growth and in vivo metastasis of breast cancer and colorectal cells [9–11]. Activation of NOTCH1 signaling has also been implicated in the severity of tumor types such as colorectal cancer [12]. Based on our finding that NOTCH1 transcriptionally activates USP11 expression, it might be interesting to study the USP11-NOTCH1 positive feedback loop in other cancer types, including colorectal and breast cancer. *Usp11* knockout mice present with normal hematopoietic system development, suggesting that USP11 inhibition might be a valid therapeutic target in cancer [5]. USP11 is a promising therapeutic target in T-ALL, as it controls the lymphocyte-specific kinase (LCK) activity downstream of the T-cell receptor (TCR) signaling pathway, blocking the activation of the glucocorticoid receptor (GR) upon application of glucocorticoids in T-ALL [5]. Mutations creating hyperactive NOTCH1 are more common in the LCK inhibitor (dasatinib)-sensitive T-ALL patient samples, suggesting that these two signaling pathways might crosstalk [13]. Additionally, the type II topoisomerase inhibitor mitoxantrone inhibits USP11 and demonstrates increased progression-free and overall survival in a relapsed T-ALL clinical trial, suggesting that USP11 inhibition can be exploited therapeutically [14, 15].

Our findings reveal a novel feedback loop regulation between NOTCH1 and USP11, underline the role of active deubiquitination in cancer, and provide a rationale for therapeutic targeting of USP11 in acute lymphoblastic leukemia.

### Supplementary information

Supplementary Fig. 1

## Data Availability

Biological material used in this study can be obtained from the authors upon request. Plasmids expressing NOTCH1 were a gift from Iannis Aifantis’ group (New York University). Plasmids expressing wild-type and catalytically inactive USP11 proteins can be provided by Addgene pending scientific review and a completed material transfer agreement. Requests for these plasmids should be submitted to Addgene and mta@addgene.org

## References

[1] Ntziachristos P, Lim JS, Sage J, Aifantis I (2014). From fly wings to targeted cancer therapies: a centennial for notch signaling. Cancer Cell.

[2] Kliza K, Husnjak K (2020). Resolving the complexity of ubiquitin networks. Front Mol Biosci.

[3] Lim R, Sugino T, Nolte H, Andrade J, Zimmermann B, Shi C (2019). Deubiquitinase USP10 regulates Notch signaling in the endothelium. Science.

[4] Jin Q, Martinez CA, Arcipowski KM, Zhu Y, Gutierrez-Diaz BT, Wang KK (2019). USP7 cooperates with NOTCH1 to drive the oncogenic transcriptional program in T-cell leukemia. Clin Cancer Res.

[5] Jin Q, Gutierrez Diaz B, Pieters T, Zhou Y, Narang S, Fijalkwoski I (2022). Oncogenic deubiquitination controls tyrosine kinase signaling and therapy response in acute lymphoblastic leukemia. Sci Adv.

[6] Wang H, Zang C, Taing L, Arnett KL, Wong YJ, Pear WS (2014). NOTCH1-RBPJ complexes drive target gene expression through dynamic interactions with superenhancers. Proc Natl Acad Sci USA.

[7] Kourtis N, Lazaris C, Hockemeyer K, Balandran JC, Jimenez AR, Mullenders J (2018). Oncogenic hijacking of the stress response machinery in T cell acute lymphoblastic leukemia. Nat Med.

[8] Yatim A, Benne C, Sobhian B, Laurent-Chabalier S, Deas O, Judde JG (2012). NOTCH1 nuclear interactome reveals key regulators of its transcriptional activity and oncogenic function. Mol Cell.

[9] Sun H, Ou B, Zhao S, Liu X, Song L, Liu X (2019). USP11 promotes growth and metastasis of colorectal cancer via PPP1CA-mediated activation of ERK/MAPK signaling pathway. EBioMedicine.

[10] Huang YY, Zhang CM, Dai YB, Lin JG, Lin N, Huang ZX (2021). USP11 facilitates colorectal cancer proliferation and metastasis by regulating IGF2BP3 stability. Am J Transl Res.

[11] Garcia DA, Baek C, Estrada MV, Tysl T, Bennett EJ, Yang J (2018). USP11 enhances TGFbeta-induced epithelial-mesenchymal plasticity and human breast cancer metastasis. Mol Cancer Res.

[12] Tyagi A, Sharma AK, Damodaran C (2020). A review on notch signaling and colorectal cancer. Cells.

[13] Gocho Y, Liu J, Hu J, Yang W, Dharia NV, Zhang J (2021). Network-based systems pharmacology reveals heterogeneity in LCK and BCL2 signaling and therapeutic sensitivity of T-cell acute lymphoblastic leukemia. Nat Cancer.

[14] Burkhart RA, Peng Y, Norris ZA, Tholey RM, Talbott VA, Liang Q (2013). Mitoxantrone targets human ubiquitin-specific peptidase 11 (USP11) and is a potent inhibitor of pancreatic cancer cell survival. Mol Cancer Res.

[15] Parker C, Waters R, Leighton C, Hancock J, Sutton R, Moorman AV (2010). Effect of mitoxantrone on outcome of children with first relapse of acute lymphoblastic leukaemia (ALL R3): an open-label randomised trial. Lancet.

