# Correction: A positive feedback loop regulation between NOTCH1 and USP11 in T-cell leukemia

**DOI:** 10.1038/s41375-023-02112-7

**Published:** 2023-12-19

**Authors:** Igor Fijalkowski, Jin Wang, Qi Jin, Jolien Van Laere, Valentina Serafin, John D. Crispino, Panagiotis Ntziachristos

**Affiliations:** 1 Department of Biomolecular Medicine, Ghent University, Ghent, Belgium; 2 Center for Medical Genetics, Ghent University and University Hospital, Ghent, Belgium; 3 Cancer Research Institute Ghent (CRIG), Ghent, Belgium; 4 Health Management Center, Renmin Hospital of Wuhan University, Wuhan, China; 5 Department of Biochemistry and Molecular Genetics, Northwestern University, Chicago, IL USA; 6 Division of Experimental Hematology, St. Jude Children’s Research Hospital, Memphis, TN USA; 7 Pediatric Research Institute Foundation “Città Della Speranza”, Padova, Italy; 8 Cellular Signaling Unit, Department of Biomedical, Metabolic and Neural Sciences, University of Modena and Reggio Emilia, Modena, Italy

**Keywords:** Leukaemia, Translational research

Correction to: *Leukemia* 10.1038/s41375-023-02096-4, published online 25 November 2023

Publisher Correction

In this article, the wrong figure and legend appeared as Fig. 1E:


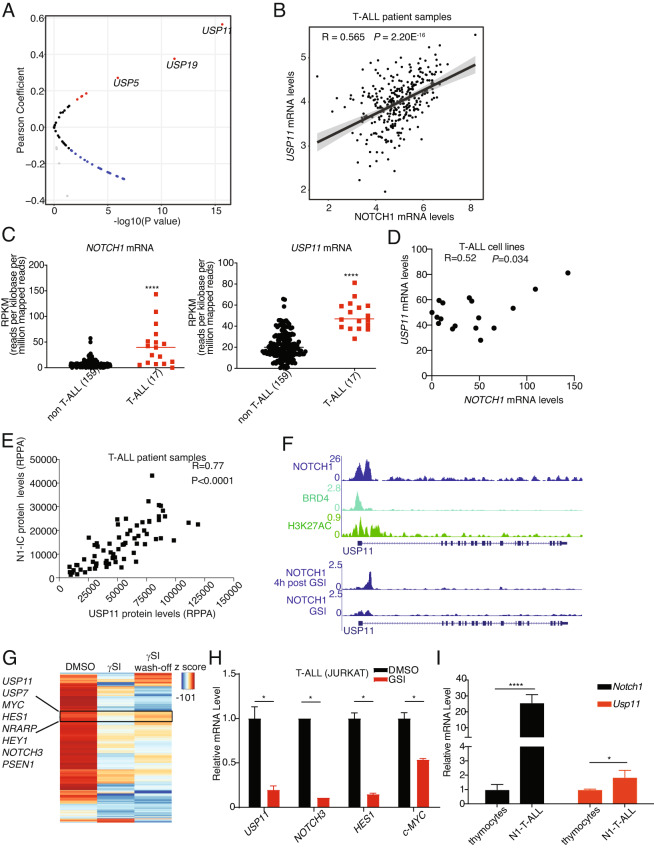


**Fig. 1** **NOTCH1 and USP11 expression levels are positively correlated in T-ALL**. **A** Volcano plot showing Pearson correlation between 52 *USPs* and *NOTCH1* mRNA levels in T-ALL patient samples analyzed from the Pediatric Cancer Genome Project data portal (PeCan, St. Jude, Memphis). **B** Pearson correlation between *NOTCH1* and *USP11* mRNA levels in T-ALL patient samples (source: PeCan). **C** RPKM values for *NOTCH1* and *USP11* in 176 blood cancer cell lines were obtained from https://software.broadinstitute.org/morpheus/, using the CCLE RNA sequencing data. These include 17 T-ALL cell lines, and all other cell lines were analyzed against these T-ALL cell lines. A two-tailed unpaired *t*-test was conducted using the RPKM values (****P < 0.0001). **D** Pearson correlation between *NOTCH1* and *USP11* mRNA levels in 17 T-ALL cell lines analyzed from CCLE RNA sequencing data. **E** Reverse-phase protein array (RPPA) for USP11 and intracellular NOTCH1 (N1IC) protein levels (*n* = 64). **F** Tracks showing NOTCH1, BRD4, and the activating histone marks H3K27Ac ChIP-Seq signal enrichment in T-ALL cells (CUTLL1) at the *USP11* locus. **G** Heatmap representation of significant gene expression changes upon treatment of CUTLL1 cells with gamma-secretase inhibitor (γSI) followed by drug wash-off for 320’ (*wash-off*) [7]. Classical NOTCH1 targets (e.g., *HES1*, *MYC*, *NRAPR*) and the deubiquitinases *USP7* and *USP11* follow the intracellular NOTCH1 levels. **H** JURKAT T-ALL cells were treated with γSI (1 μM) for 24 h. RT-qPCR analysis of *USP11* and other *NOTCH1* targets was shown (*P < 0.05). **I** RT-qPCR analysis of *Usp11* and *Notch1* in normal mouse thymocytes and spleen cells isolated from N1ΔE induced mouse T-ALL model (*P < 0.05, ****P < 0.00001).

The figure and legend should have appeared as shown below:


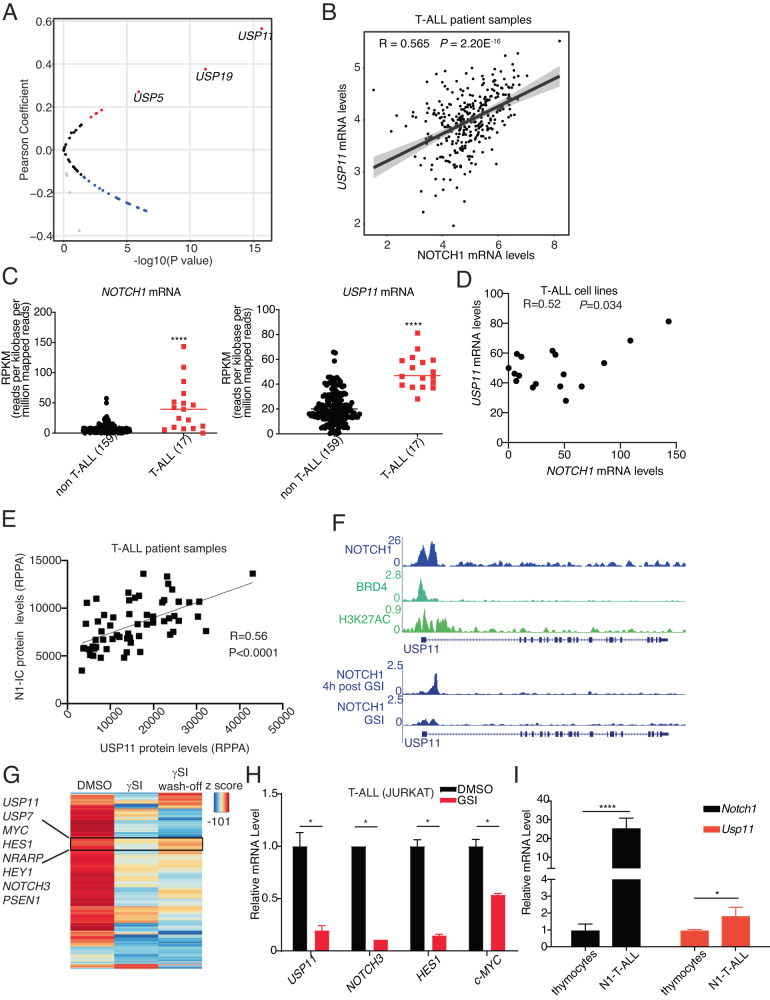


**Fig. 1 NOTCH1 and USP11 expression levels are positively correlated in T-ALL.** **A** Volcano plot showing Pearson correlation between 52 *USPs* and *NOTCH1* mRNA levels in T-ALL patient samples analyzed from the Pediatric Cancer Genome Project data portal (PeCan, St. Jude, Memphis). **B** Pearson correlation between *NOTCH1* and *USP11* mRNA levels in T-ALL patient samples (source: PeCan). **C** RPKM values for *NOTCH1* and *USP11* in 176 blood cancer cell lines were obtained from https://software.broadinstitute.org/morpheus/, using the CCLE RNA sequencing data. These include 17 T-ALL cell lines, and all other cell lines were analyzed against these T-ALL cell lines. A two-tailed unpaired *t*-test was conducted using the RPKM values (****P < 0.0001). **D** Pearson correlation between *NOTCH1* and *USP11* mRNA levels in 17 T-ALL cell lines analyzed from CCLE RNA sequencing data. **E** Reverse-phase protein array (RPPA) for USP11 and intracellular NOTCH1 (N1IC) protein levels (*n* = 61). **F** Tracks showing NOTCH1, BRD4, and the activating histone marks H3K27Ac ChIP-Seq signal enrichment in T-ALL cells (CUTLL1) at the *USP11* locus. **G** Heatmap representation of significant gene expression changes upon treatment of CUTLL1 cells with gamma-secretase inhibitor (γSI) followed by drug wash-off for 320’ (*wash-off*) [7]. Classical NOTCH1 targets (e.g., *HES1*, *MYC*, *NRAPR*) and the deubiquitinases *USP7* and *USP11* follow the intracellular NOTCH1 levels. **H** JURKAT T-ALL cells were treated with γSI (1 µM) for 24 h. RT-qPCR analysis of *USP11* and other NOTCH1 targets was shown (*P < 0.05). **I** RT-qPCR analysis of *Usp11* and *Notch1* in normal mouse thymocytes and spleen cells isolated from N1ΔE induced mouse T-ALL model (*P < 0.05, ****P < 0.00001).

The original article has been corrected.

