# Supplementary material for: A positive feedback loop regulation between NOTCH1 and USP11 in T-cell leukemia
Source: Leukemia. 2023 Nov 25;38(1):193–7. doi: 10.1038/s41375-023-02096-4 (PMC10776390; doi:10.1038/s41375-023-02096-4)
Supplement: Supplementary file 1 — Supplementary Fig. 1 [file 41375_2023_2096_MOESM1_ESM.pdf]

Supplementary Figure 1

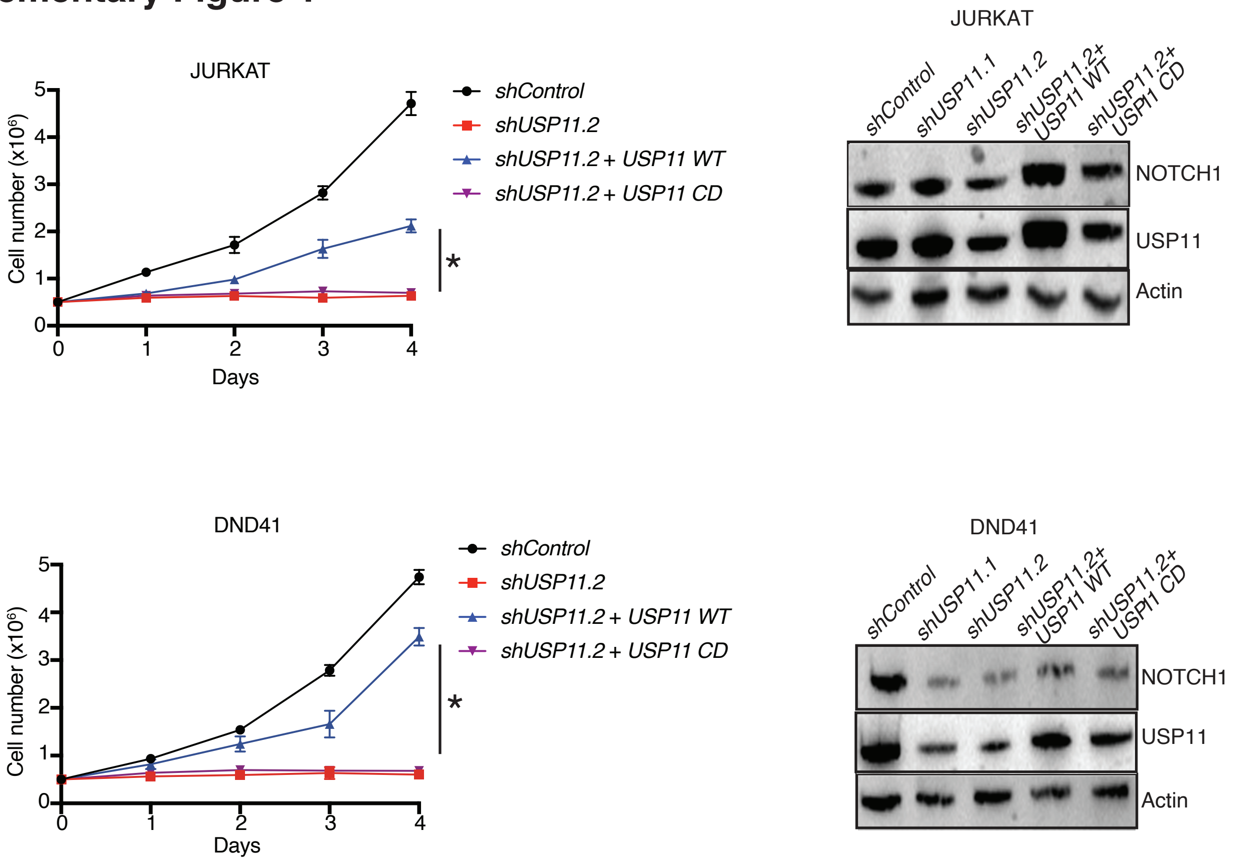

Supplementary Figure 1. Ectopic expression of the catalytically active (WT) USP11 rescues shUSP11-mediated inhibition of T-ALL cell growth. Growth studies over 4 days of wild-type (WT) and catalytically inactive (CD) USP11-expressing JURKAT (upper panel) and DND41 (lower panel) cells transduced with control or shUSP11.2 lentivirus (n=3 biological replicates, * P≤0.05, left panels). Immunoblot detection of NOTCH1, USP11, and GAPDH protein levels (day 0) in cells used in the growth studies (right panels).
